# Supplementary material for: Phytobacter diazotrophicus from Intestine of Caenorhabditis elegans Confers Colonization-Resistance against Bacillus nematocida Using Flagellin (FliC) as an Inhibition Factor
Source: Pathogens. 2022 Jan 10;11(1):82. doi: 10.3390/pathogens11010082 (PMC8778419; doi:10.3390/pathogens11010082)
Supplement: Supplementary file 1 [file pathogens-11-00082-s001.zip › pathogens-1507418-supplementary.pdf]

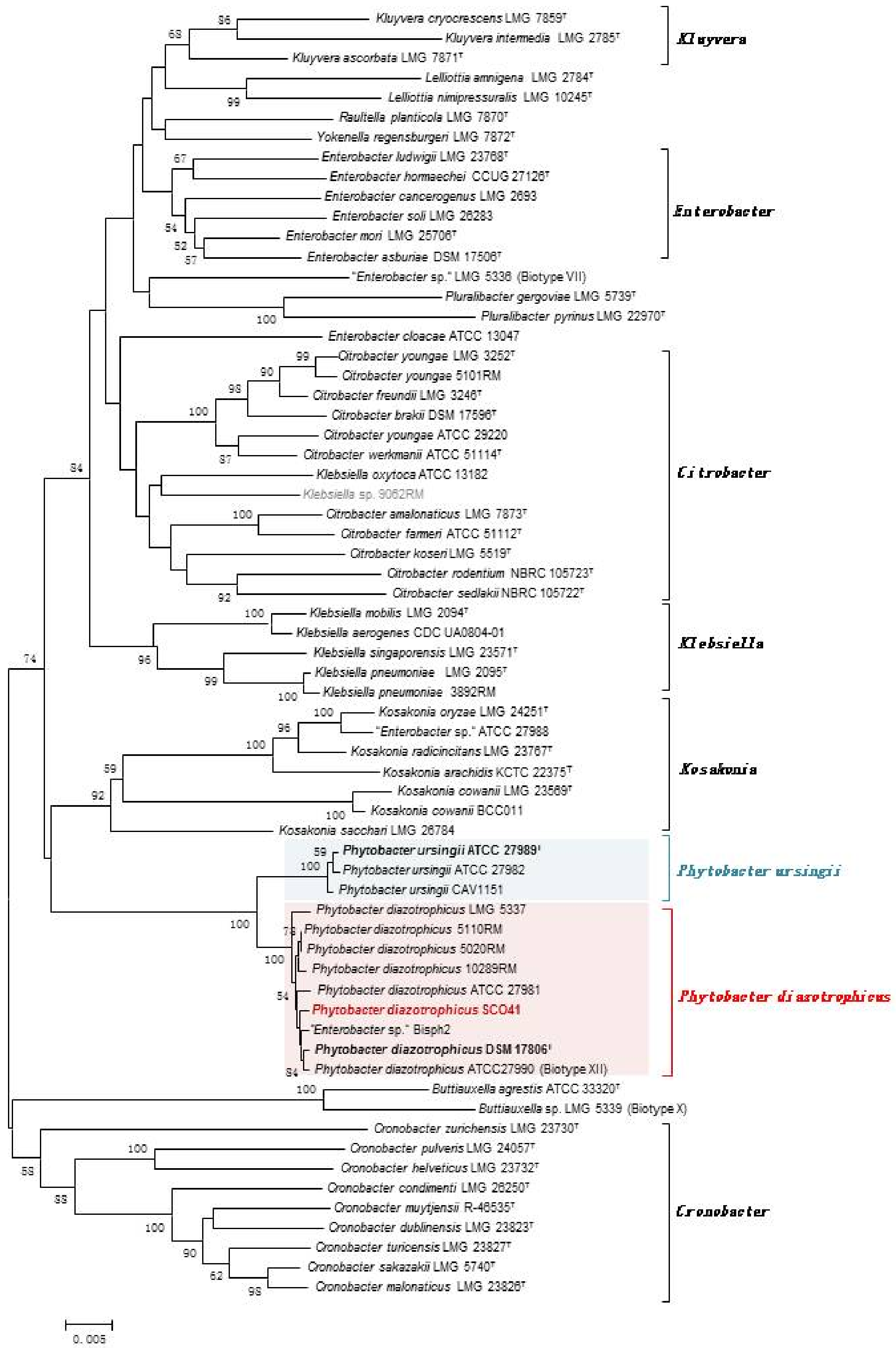

Figure S1. Neighbor-joining unrooted tree based on the concatenated partial *atpD*, *gyrB*, *infB* and *rpoB* gene sequences (2548 bp), depicting the relationships of *P. diazotrophicus* SCO41 and its nearest phylogenetic relatives. Bootstrap values of above 50 % are given at nodes based on 1000 replications. Bar, 1 % nucleotide substitutions. The superscript letters (T) represent typical strains of the species.

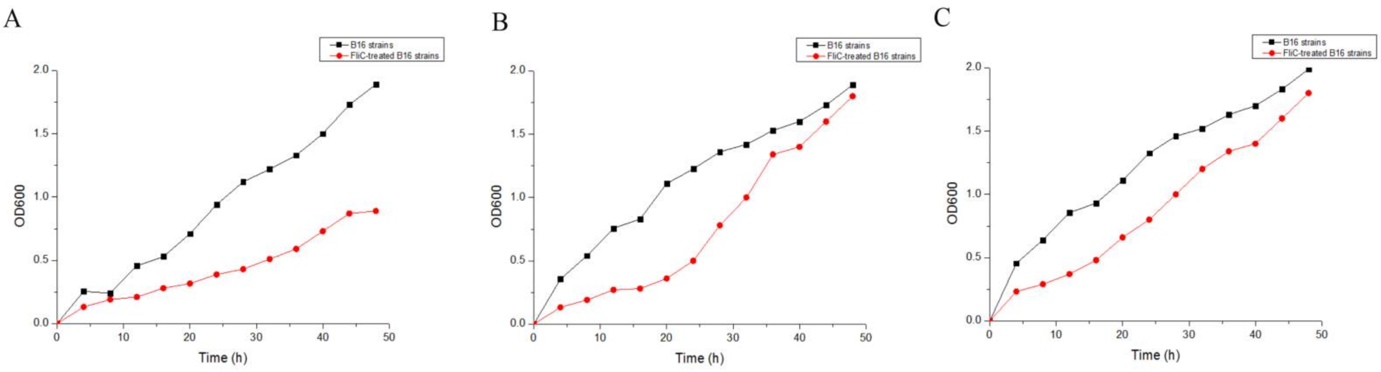

Figure S2. Results of the stress tolerance assays experiments. (**A**) Growth status of bacteria under heat stress; (**B**) Growth status of bacteria under drought stress; (**C**) Growth status of bacteria under H_2O_2 stress.

Table S1. Interface amino acid residues between FliC and serine/threonine protein kinase of *B. nematocida* B16

FliC	Serine/Threonine Protein Kinase
A: LEU9	A: VAL186
A: LEU9	A: LEU187
A: LEUl2	A: LEU187
A: LEU17	A: PHE400
A: LYS19	A: lLE404
A: SER20	A: PHE400
A: ALA23	A: PHE400
A: ALA23	A: LEU403
A: ALA23	A: ILE404
A: ARG30	A: LEU403
A: ARG30	A: PHE411
A: ARG30	A: lLE414
A: ARG36	A: PHE411
A: ASN38	A: LYS408
A: ALA40	A: PHE411
A: ALA40	A: LEU415
A: LYS41	A: LEU415
A: SER451	A: PRO422
A: ARG452	A: ILE419
A: ARG452	A: lLE423
A: ASP455	A: LEU415
A: ASP457	A: LEU418
A: THR460	A: TRP81
A: GLU461	A: LEU418
A: GLU461	A: ILE421
A: SER463	A: TRP81

A: ASN464	A: TRP81
A: ASN464	A: LEU85
A: MET465	A: PHE356
A: MET465	A: ILE414
A: ARG467	A: LEU82
A: ARG467	A: PHE356
A: ARG467	A: LEU360
A: ALA468	A: PHE356
A: LEU471	A: ILE363
A: GLN472	A: PHE400

Table S2. Interface amino acid residues between FliC and membrane protein insertase YidC of *B. nematocida* B16.

FliC	Membrane Protein Insertase YidC
A: THR6	B: TRP47
A: ASN7	B: TRP47
A: SER10	B: TRP47
A: ASN16	B: PHE40
A: LYS19	B: PHE40
A: ARG467	B: PHE140
A: LEU471	B: PHE140
A: ALA474	B: ILE141
A: VAL478	B: LEU75
A: GLN481	B: ALA74
A: GLN481	B: LEU78
A: GLN481	B: PRO77

Table S3. Interface amino acid residues between FliC and micro-aerobic cytochrome bd terminal oxidase CydB of *B. nematocida* B16 (small red box in Figure 10).

FliC	Membrane Protein
A: GLN21	C: PHE766
A: LEU24	C: TYR770
A: GLY25	C: TYR770
A: ILE28	C: TYR770
A: ILE470	C: LEU767
A: ILE470	C: VAL774
A: GLN473	C: ALA771
A: THR476	C: PHE766

Table S4. Interface amino acid residues between FliC and micro-aerobic cytochrome bd terminal oxidase CydB of *B. nematocida* B16 (big red box in Figure 10).

FliC	Membrane Protein
A: SER64	B: PHE267
A: ARG65	B: PHE267
A: ARG65	B: PHE267
A: ASN68	B: ILE263
A: ASN68	B: PHE267
A: ASP69	B: PHE244
A: ASP69	B: PHE267
A: ILE71	B: TRP260
A: ILE71	B: ILE263
A: ILE71	C: ALA777
A: ILE71	C: LEU778
A: SER72	B: PHE244
A: SER72	B: ILE247
A: GLN75	B: ILE247
A: GLN75	B: LEU251
A: GLN75	B: TRP260
A: THR76	B: IL E247
A: GLU78	B: TRP250
A: ASN82	B: TRP250
A: ASN82	B: ARG254
A: GLN130	B: LEU243
A: PHE131	B: ILE240
A: PHE131	B: LEU243
A: PHE131	B: PHE244
A: ASN132	B: ILE240
A: GL N429	B: TRP260
A: ASN430	B: TRP260
A: ASN433	B: TRP260
A: ASN444	B: TYR320
